# Temporal Trends in Fecal Occult Blood Test: Associated Factors (2009–2017)

**DOI:** 10.3390/ijerph16122120

**Published:** 2019-06-14

**Authors:** Ana Isabel Cobo-Cuenca, José Alberto Laredo-Aguilera, María-Aurora Rodríguez-Borrego, Esmeralda Santacruz-Salas, Juan Manuel Carmona-Torres

**Affiliations:** 1 Departamento de Enfermería, Fisioterapia y Terapia Ocupacional, Universidad de Castilla la Mancha (UCLM), 45071 Toledo, Spain; anaisabel.cobo@uclm.es (A.I.C.-C.); esmeralda.santacruz@uclm.es (E.S.-S.); Juanmanuel.carmona@uclm.es (J.M.C.-T.); 2 Grupo de Investigación Multidisciplinar en Cuidados (IMCU), UCLM. Av. Carlos III s/n., 45071 Toledo, Spain; 3 Instituto Maimónides de Investigación Biomédica de Córdoba (IMIBIC), 14004 Córdoba, Spain; en1robom@uco.es; 4 Departamento de Enfermería. Universidad de Córdoba (UCO), 14004 Córdoba, Spain

**Keywords:** colorectal cancer, cancer screening, Spain, public health

## Abstract

A cross-sectional study with 27,821 records of non-institutionalized people in Spain aged between 50–69 years old (59.94 ± 5.8 years), who participated in the European Health Survey in Spain (2009, 2014) and National Health Survey (2011/12, 2017). Fecal occult testing, the reason for performing the test, age, sex, nationality, social status, marital status, education level, body mass index (BMI), and place of residence. Overall, 54% were women, 93.9% were Spanish, 47.8% had a secondary study, and 66.4% were married. Across the years, the rate of the fecal occult blood test (FOBT) increased significantly (*p* < 0.001). This increase can be accounted for a letter campaign advising testing (45%, *p* < 0.001). FOBT was associated with more age (odds ratio—OR 1.04, 95% confidence interval—CI 1.04–1.05, *p* < 0.001), Spanish nationality (OR 1.91, 95% CI 1.25–2.93, *p* = 0.003), being married (OR 1.13, 95% CI 1.02–1.25, *p* = 0.025), having a higher level of education (OR 2.46, 95% CI 2.17–2.81, *p* < 0.001), belonging to high social classes (OR 1.35, 95% CI 1.12–1.64, *p* = 0.001), and BMI <25 (OR 1.72, 95% CI 1.25–2.37). Frequency of FOBT has increased in recent years. Performing FOBT is associated with age, nationality, marital status, higher education level, and social class.

## 1. Introduction

Worldwide, colorectal cancer (CRC) is the third most commonly diagnosed cancer in men and the second most common in women. The incidence of CRC is greatest in developed countries [1,2,3]. In recent years, the incidence of CRC has been increasing in Europe and Africa [2]. It is estimated that the incidence of CRC will increase by more than 60% by 2030 [4], possibly due to an increase in risk factors such as smoking, obesity, a non-healthy diet, and alcohol [4,5,6].

To prevent deaths from CRC, it is necessary to reduce the associated risk factors [6] and to develop policies for early detection [7,8].

The fecal occult blood test (FOBT) is recommended for CRC screening. The FOBT is popular because it is inexpensive and without risk [9]. If the FOBT is positive, a colonoscopy under sedation is performed. FOBTs are performed more than colonoscopies because the FOBT is a non-invasive test and does not cause pain [10,11].

The FOBT can be performed by two different analytical methods: Guaiac-FOBT and a fecal immunochemical test (FIT). Although the Guaiac-FOBT has been widely used for CRC screening, the FIT is more sensitive than the Guaiac-FOBT for detecting colorectal cancer [12].

In Europe [3,8] and specifically in Spain [13,14], the incidence of CRC is increasing. Following the recommendations of the European Screening Guideline for CRC [15] and the cancer strategy of the National Health System [16], Spain started CRC screening programs for people aged 50–69 years. First, people received an invitation letter every two years asking them to take the FOBT and, if the test were positive, a colonoscopy was performed under sedation [7,17]. In Spain, there are 17 autonomous communities. Although the national health system is public, its management is different in each autonomous community; it is dependent on the regional government. For this reason, the implementation of CRC screening has been disparate in different communities. As of 2017, CRC screening had not been fully established in all Spanish territories.

Therefore, it would be interesting to know the frequency of FOBT, the reasons why it is performed, and the sociodemographic variables that might be associated with its use. This information would likely help to increase the effectiveness of CRC detection programs.

The aims of this study were to determine the prevalence and temporal trends of FOBT in people aged 50–69 years, and to determine the sociodemographic profiles and the associated variables with participation in CRC screening.

## 2. Materials and Methods 

### 2.1. Participants and Design

In this cross-sectional study, the population consisted of non-institutionalized people aged 50–69 years. They resided in Spain, having participated in the European Health Survey in Spain (EHSS) in 2009 [18], and 2014 [19], and in the National Health Survey (NHS) 2011/12 [20], and 2017 [21]. These surveys were carried out by the National Institute of Statistics (NIS) and the Spanish Ministry of Health, Social Services, and Equality (SMHSE).

The EHSS and NHS were carried out through a personal interview executed by the NIS and SMHSE, using a probabilistic multi-stage sampling with stratification of first- (municipalities) and second-stage (sections) units, with the final units (individuals) by random routes and sex- and age-based quotas. The data obtained from these surveys are available in the NIS and SMHSE websites [18,19,20,21] in the form of anonymized microdata, so no special authorizations are required for their use. Since we used this type of data for the present study, an ethics committee report was unnecessary, according to Spanish law.

For the current study, all records for people aged 50–69 years were selected. The sample totaled 27,821 records: 6361 from year 2009; 6252 from years 2011/12; 7146 from year 2014; and 8042 from year 2017.

### 2.2. Outcomes Measures

The data collection instruments used by the NIS and SMHSE were the 2009, and 2014 EHSS and the 2011/12, and 2017 NHS [18,19,20,21]. In these surveys, participants were asked about various preventive health practices, such as FOBT.

The dependent variables in the study were the frequency of performing FOBT and the reason for performing the test.

The independent variables in the study were the year of study and sociodemographic variables: Sex, age, autonomous community, nationality, marital status, educational level, and social class. Social class was stratified into three levels: High class (level I: Directors and managers of companies with 10 or more employees and professionals with university degrees; level II: Directors of companies with less than 10 employees and professionals with college diplomas); medium class (level III: Intermediate occupations; level IV: Workers in qualified technical occupations); and low class (level V: Primary sector workers, level VI: Unskilled workers). Social class was established according to the categories proposed by the Spanish Society of Epidemiology [22]. Regarding health status, we included the variables of body mass index (BMI), which was calculated from self-reported body weight and height and categorized according to the World Health Organization (WHO) [23], self-perceived health status, diseases suffered in the last 12 months (chronic constipation or hemorrhoids), and existence of chronic or long-term illness.

### 2.3. Statistical Analysis

For a descriptive analysis of the quantitative variables, the mean (m) and standard deviation (SD) were calculated. For a descriptive analysis of qualitative variables, count (*n*) and proportions (%) were used. We also compared proportions of categorical variables using chi-squared tests for contingency tables. Multiple logistic regression was also performed to determine the influence of the variables in the performance of FOBT. We used the Wald statistic, in which the variables with *p* ≥ 0.15 were eliminated one-by-one from the model. The odds ratios (ORs) were calculated with their confidence intervals. All the contrasts of hypotheses were bilateral, and statistical significance was established at *p* < 0.05. Data analysis was performed using the statistical program IBM SPSS Statistics version 24 (IBM Corp, Armonk, NY, USA), licensed to the University of Castilla-La Mancha (UCLM).

### 2.4. Ethics Statement

The data obtained from these surveys are available in the NIS and SMHSE website: www.ine.es in the form of anonymized microdata, so no special authorizations are required for their use. Since we used this type of data for the present study, an ethics committee report was unnecessary, and no authorization for its use is required, according to Spanish law. To anyone interested, the records are accessible on the INE website in the form of an anonymous microdata file.

## 3. Results

A total of 27,821 records of people aged 50–69 years who participated in NHS 2011/12 and 2014; and EHSS 2009 and 2017 were analyzed. Among the participants, 54.2% were female, with a mean age of 59.4 ± 5.8 years. The most frequent sociodemographic characteristics were that they were married (66.4%), had Spanish nationality (94%), had secondary or professional training education (47.8%), and had a good self-perceived health status (49.9%). Table 1 shows the sociodemographic characteristics of the participants according to the year of the interview.

In general, there was a significant increase in FOBT from 2009 to 2017 (8.5% vs. 31.8%; *p* < 0.001). There was also significant differences in the reason for FOBT (*p* < 0.001). Receipt of a letter advising testing increased the testing from 26.6% in 2011/12 to 45% in 2017 (Table 2). There were significant differences in the proportions of those reporting receipt of a letter advising testing as the reason for volunteering for FOBT.

Table 3 shows the trend in FOBT in the different autonomous communities of Spain from 2009 to 2017. In 2014, only La Rioja, Cantabria, and País Vasco (41.5%) were the autonomous communities with the highest percentage of FOBT (*p* < 0.001). In 2017, in the majority of the communities, the percentage of testing had increased to >50%. 

The logistic regression analysis (Table 4) shows that FOBT was associated mainly with age (OR 1.4, 95% CI 1.04–1.05, *p* < 0.001), having Spanish nationality (OR 1.91, 95% CI 1.25–2.93, *p* = 0.003), being married (OR 1.13, 95% CI 1.02–1.25, *p* = 0.025), secondary-level education (OR 2.46 95% CI 2.17–2.81, *p* < 0.001), university level of education (OR 2.31, 95% CI 1.99–2.67, *p* < 0.001), belonging to social classes I or II (OR 1.35, 95% CI 1.12–1.64, *p* = 0.002), and having normal weight (relative to overweight/obesity; OR 1.72, 95% CI 1.25–1.37, *p* = 0.001).

## 4. Discussion

In Europe, CRC screening programs were first implemented in the year 2000, but their use has been dissimilar among the different countries. For example, France, Finland, United Kingdom, and Slovenia have fully developed population screening, whereas, in Spain, Belgium, Holland, Poland, Malta, and Italy, the CRC population screening has just now been implemented. In Portugal, Norway, and Sweden, CRC population screening remains in a pilot phase [24,25].

In Spain, Catalonia was the first autonomous community to carry out screening programs with a pilot study in 2000 [26]. In 2009, the Health, Social Services, and Equality Ministry recommended CRC screening programs with FOBT every two years for people 50–69 years-old; this was to be done for at least 50% of this population by the year 2015 and for 100% by the year 2025 [16]. This implementation has progressed gradually. In 2010, screening was only established in six of the 17 autonomous communities (Catalonia 2000, Valencia 2005, Murcia 2006, Cantabria 2008, Canarias 2009, and País Vasco 2009) [27]. In 2017, the CRC screening had been implemented in 11 autonomous communities and was being introduced in another five. Now (2018), the rest of the communities have just implemented the colorectal cancer screening (except Ceuta and Melilla). This unequal implementation was due to each region having one public health system that is managed by a different regional government, even though the health system in Spain is public.

Our study shows how the frequency of FOBT participation has increased between the years 2009 and 2017. Furthermore, the reason why FOBT is performed in people aged 50–69 years has changed. The FOBT was more frequently performed because people had received an invitation letter, rather than because of clinical symptoms. Screening implementation in Spain was unequal; for example, in Catalonia, 14.4% of the population was adherent to CRC screening in 2008 [28], with increasing participation rates (35%–66%) in some autonomous communities in the following years. This includes Pais Vasco, Murcia, Valencia, Canarias, Catalonia, Cantabria, Aragon, and Albacete [17,29,30,31,32].

The rate of population screening for CRC usually ranges from 42% to 47%, depending on the type of test used (guaiac-FOBT or FIT) [11]. Currently, in Spain, the main screening program uses FIT [25]

The European Guidelines for Quality Assurance in CRC Screening and Diagnosis (2010) hoped to achieve the desired implementation rates ≥65%, with rates ≥45% considered acceptable [33].

In this study, in 2014, 21% of Spanish people between 50–69 years had performed the FOBT. Although this rate is relatively low, it has increased in recent years. This might be because CRC screening had not been fully established in the health services of each autonomous community. However, in 2017 the rate increased, 31% of Spanish people aged between 50–69 years had performed the FOBT. Although this rate is considered unacceptable [33], these rates varied in different autonomous communities. In 2017, only six communities achieved unacceptable rates (Andalucía, Asturias, Extremadura, Madrid, Murcia, and Ceuta-Melilla), as they were just starting the implementation of the screening program. In contrast, the rest of the communities have achieved rates close to 60% and three have achieved over 65%.

In this study, the sociodemographic variables that are most associated with FOBT participation are higher education, higher socioeconomic level, being married, and age [11,34,35]. These variables are consistent with other screening programs carried out in Spain [36,37].

Even though the Spanish health system is public, lower realization of FOBT is associated with low socioeconomic status, low level of education, and being a foreigner. This might be due to lower access to the health system, lack of knowledge about colorectal cancer and its prevention, language barriers, and cultural differences.

Therefore, for screening to reach these demographics, it is important to consider the previously identified factors [38,39,40].

On the other hand, a higher BMI is associated with non-adherence to FOBT in people aged 50–69 years. This correlates with other studies in which lifestyles and weight were associated with non-participation in screening programs [41]. This might be because people with normal weight are more aware of their health status and have greater participation in practices for conserving and improving their health.

In other studies, women have greater adherence to CRC screening than men [11,17,42]. This adherence increases in women who also participate in screening programs of breast and cervical cancer [35,43]. In this study the women and men participated in FOBT similarly, as with García et al. [34]. To improve female participation, Bocci et al. (2015) suggested giving the FOBT kit to women while they are attending mammography or gynecological examinations [43].

It has been previously shown that inclusion of the FOBT kit in the invitation letter increases the probability of participation [32,35,42]. The participation rate increases if a second reminder letter is sent [35,39]. Therefore, the difference in adherence in the different screening programs might be due to variations in the invitation, public awareness campaigns, population types, and implementation periods [11].

The medical recommendation for screening remains is still one of the main reasons for testing. Primary care professionals (physicians and nurses) play an important role in the prevention of cancer, through providing information, promoting healthy lifestyles, and early detection methods [7,17,44].

The publicity for screening programs in Spanish autonomous communities has led to more general knowledge about the significance of early detection of CRC [11,17,31,34,43,44,45,46,47].

### Strength and Limitations

Since we have used secondary data (microdata) obtained by the NIS, we cannot know the type of fecal test that was utilized for CRC screening (Guaiac-FOBT or FIT). This question was not included in the NHS and EHSS surveys, although currently, in Spain the majority of screening programs use FIT. Although this is a limitation, we have reached our aim of determining the temporal trend in the implementation of FOBT in Spanish people and what factors influence adherence. Another limitation was that the analyzed data are self-reported information. Another limitation was that we use data from the NHSS and EHSS, which are sectional-cross studies, and we cannot determine the association of variables or causality.

On the other hand, the study has some strengths because it utilizes a large updated sample that is representative at the national level.

## 5. Conclusions

The rate of FOBT increased between the years 2009 and 2017. The implementation of CRC screening in Spain has gradually increased. In 2010, only six of 17 regional health services had implemented CRC screening by FOBT. However, in 2017 only six autonomous communities had rates <45%, due to a retard in implementing screening. The profile of Spanish people aged 50–69 years who participated less in the FOBT was middle-aged, low socioeconomic level, low educational level, BMI >25, and being a foreigner. The main reason for fecal occult blood testing in people aged 50–69 years was having received an invitation letter. Overall, the increasing participation rate seems to indirectly indicate the effectiveness of screening programs in the different autonomous communities of Spain. An organized program of colorectal screening, with regularly scheduled invitations to screening and adequate follow-up, will result in the greatest impact of a screening program directed against CRC.

Highlights:

Variables associated with performing FOBT are higher educational, social class, civil status, and BMI ≤25.

There are differences in the prevalence of FOBT testing according to different health services.

Prevalence rates of testing FOBT from 2009 to 2017 have been increased in the Spanish population.

## Figures and Tables

**Table 1:** Sociodemographic characteristics of Spanish people 50–70 years (*n* = 27,821) in the period 2009–2017.

Characteristics *n* (%)	2009 *n* = 6381 (%)	2011/12 *n* = 6252 (%)	2014 *n* = 7146 (%)	2017 *n* = 8042 (%)	*p*
*Sex*					
Men	2871 (45)	2902 (46.4)	3338 (46.7)	3829 (47.6)	
Women	3510 (55)	3350 (53.6)	3808 (53.3)	4213 (52.4)	0.107
*Nationality*					
Spanish	6197 (97)	6045 (96.7)	6931 (97)	27,726 (96.1)	0.358
Foreigner	184 (3)	207 (3.3)	215 (3)	316 (3.9)	
*Marital status*					
Single	748 (11.7)	784 (12.6)	961 (13.5)	1103 (13.7)	<0.001
Married	4335 (67.9)	4182 (66.9)	4655 (65.2)	5301 (65.9)	
Widowed	752 (11.9)	688 (11)	751 (10.6)	707 (8.8)	
Separated	244 (3.8)	255 (4.1)	279 (3.9)	303 (3.8)	
Divorced	297 (4.7)	335 (5.4)	486 (6.8)	603 (7.6)	
*Level of education*					<0.001
Without studies	1231 (19.3)	NR	741 (10.3)	717 (8.9)	
Primary	2295 (36)		2294 (32.1)	1872 (23.3)	
Secondary/PT	1992 (31.2)		2933 (41)	4124 (51.3)	
University	856 (13.5)		1178 (16.6)	1329 (16.5)	
*Social Class*					
Class I and II	NR	1160 (19.1)	1407 (20)	1376 (17.4)	0.348
Class III and IV		2104 (34.7)	2439 (34.8)	2752 (34.9)	
Class V and VI		2802 (46.2)	3173 (45.2)	3764 (47.7)	
*Body Mass Index*					
Insufficient	32 (0.5)	48 (0.8)	65 (0.9)	82 (1)	<0.001
Normal weight	1924 (30.2)	1848 (29.6)	2387 (33.4)	2657 (33.0)	
Overweight	2696 (42.3)	2507 (40.1)	2910 (40.7)	3354 (41.7)	
Obesity	1324 (20.8)	1358 (21.7)	1508 (21.1)	1667 (20.7)	
No answer	405 (6.2)	491 (7.8)	276 (3.9)	282 (19.4)	
*Self-perceived health status*					
Very good	608 (9.5)	703 (11.2)	831 (11.5)	963 (12)	
Good	3095 (48.5)	3124 (50)	3554 (50)	4112 (51.1)	<0.001
Regulate	1836 (28.8)	1709 (27.3)	1926 (27)	2136 (26.6)	
Bad	650 (10.2)	590 (9.4)	609 (8.5)	646 (8)	
Very bad	192 (3)	126 (2)	226 (3.1)	185 (2.3)	
Hemorrhoids*					
Yes	NR	502 (66.7)	558 (63)	582 (69.1)	<0.031
No		251 (33.3)	326 (37)	260 (30.9)	
Constipation*					
Yes	NR	393 (92)	403 (91.6)	359 (91.1)	<0.715
No		34 (8)	36 (8.4)	35 (8.9)	
*Chronic or long-term illness*					
Yes	4351 (68.2)	3641(58.2)	5417 (76)	6274 (78)	<0.001
No	2025 (31.8)	2606 (41.8)	1725 (24)	166 (22)	

PT, professional training; * In the last 12 months; NR: Not registered.

**Table 2:** Fecal occult blood testing of Spanish people between 50–70 years (*n* = 27,821) in the period 2009–2017.

Characteristics	2009 *n* = 6381 (%)	2011/12 *n* = 6252 (%)	2014 *n* = 7146 (%)	2017 *n* = 8042 (%)	*p*
Conducting FOB * test					
Yes	544 (8.5%)	661 (10.6%)	1502 (21%)	2559 (31.8%)	<0.001
No	5753 (90.2%)	5474 (87.6%)	5576 (78%)	5445 (67.7%)	
Do not answer	84 (1.3%)	117 (1.8%)	68 (1%)	38 (0.5%)	
Reason for FOB* test					
For problem, symptom or illness	Not registered	284 (43%)	529 (35.2%)	1345 (28.5%)	<0.001
On the advice of your primary care physician or specialist, although you had no problem		148 (22.4%)	306 (20.4%)	1072 (22.7%)	
Because you received a letter, someone called you, or you were asked at your health center if you wanted to do this test		176 (26.6%)	598 (39.8%)	2126 (45%)	
Other reasons		49 (7.4%)	66 (4.4%)	164 (7.6%)	
Do not answer		4 (0.6%)	3 (0.2%)	3(0.1%)	
*Testing frequency*					
Two years or less	221 (40.6%)	415 (62.8%)	968 (64.5%)	1945 (75.8%)	<0.001
Between two and three years	59 (10.8%)	68 (10.3%)	164 (10.9%)	238 (9.3%)	
More than three years	261 (48%)	174 (26.3%)	364 (24.2%)	369 (14.4%)	
Do not answer	3 (0.6%)	4 (0.6%)	6 (0.4%)	7 (0.3%)	

FOB*: Fecal occult blood.

**Table 3:** Fecal occult blood testing of Spanish people between 50–70 years *(n =* 27,821) in the period 2009–2017 in the Communities’ Spain.

Communities´Spain	2009	2011	2014	2017	*p*
	FOB	FOB	FOB	FOB	
	*n* (%)	*n* (%)	*n* (%)	*n* (%)	
Andalucía	21(12.7%)	27 (16.4%)	44 (26.7%)	73 (44.2%)	<0.001
Aragón	8 (6.5%)	13 (10.5%)	18 (15.5%)	85 (68.5%)	
Asturias	8 (14.8%)	9 (16.7%)	14 (25.9%)	23 (42.6)	
Baleares	10 (10.5%)	8 (8.4%)	20 (21.1%)	57 (60%)	
Canarias	12 (5.6%)	31 (14.1%)	54 (25%)	119 (55.1%)	
Cantabria	8 (3.7%)	7 (3.2%)	84 (38.5%)	119 (54.6%)	
Castilla y León	12 (5.1%)	14 (5.9%)	31 (13.1%)	180 (75.9%)	
Castilla la Mancha	7 (9%)	8 (10.3%)	13(16.7%)	50 (64.1%)	
Cataluña	17 (5.7%)	27 (9.1%)	79 (26.7%)	173 (54.4%)	
C. Valenciana	26 (6.1%)	51 (11.9%)	106 (24.8%)	245 (57.2%)	
Extremadura	9 (17%)	9 (17%)	13 (24.5%)	22 (41.5%)	
Galicia	15 (8.9%)	24 (14.2%)	43 (25.4%)	87 (51.5%)	
Madrid	23 (16.7%)	16 (11.6%)	42 (30.4%)	57 (41.3%)	
Murcia	15 (7.6%)	43 (21.7%)	64 (32.3%)	76 (38.4%)	
Navarra	3 (1.4)	6 (2.8%)	36 (16.9%)	168 (78.9%)	
País Vasco	20 (2.9%)	103 (15%)	236 (34.4%)	328 (47.7%)	
La Rioja	1 (0.7%)	14 (9.2%)	64 (41.8%)	74 (48.4%)	
Ceuta y Melilla	6 (22.2%)	5 (18.5%)	7 (25.9%)	9 (33.3%)	

**Table 4:** Logistic regression model for the association among sociodemographic characteristics and fecal occult blood test in Spanish people aged between 50–69 years (2009–2017).

	OR (95% CI)	*p*
*Age Group*	1.04 (1.04–1.05)	<0.001
*Nationality*		
Spanish	1.91 (1.25-2.93)	0.003
Foreigner	Reference	
*Marital status*		
Single	Reference	
Married	1.13 (1.02–1.25)	0.025
Widowed/Separated/Divorced	1.03 (0.91–1.16)	0.647
*Level of education*		
Without education	Reference	
Primary	1.52 (1.33–1.73)	<0.001
Secondary or PT	2.46 (2.17–2.81)	<0.001
University	2.31 (1.99–2.67)	<0.001
*Social class*		
Class I and II	1.35 (1.12–1.64)	0.002
Class III and IV	1.33 (1.16–1.52)	0.001
Class V and VI	Reference	
*Body mass index*		
Insufficient	1.05 (0.98–1.137)	0.119
Normal weight	1.72 (1.25–2.37)	0.001
Overweight/obesity	Reference	

OR: Odds ratio; CI 95%: 95% confidence interval.
